# Supplementary material for: Decreased Glucocorticoid Receptor Expression and Function in Cord Blood Immune Cells from Preterm Neonates with Morbidity
Source: Int J Mol Sci. 2025 Nov 3;26(21):10686. doi: 10.3390/ijms262110686 (PMC12608741; doi:10.3390/ijms262110686)
Supplement: Supplementary file 1 [file ijms-26-10686-s001.zip › ijms-3867359-SI.pdf]

Supplementary Table S1: Real-time SYBR green and Probe PCR primers.

Gene	Primers /Probe	Sequence	Annealing temp (ºC)	Extension time	Accession number
ACTB	Forward	AGAAAATCTGGCACCACACC	55	18s	NM_001101.5
	Reverse	TAGCACAGCCTGGATAGCAA			
IL6	Forward	GGTACATCCTCGACGGCATCT	56	10s	NM_000600.5
	Reverse	GTGCCTCTTTGCTGCTTTCAC			
ICAM1	Forward	CCTTCCTCACCGTGTACTGG	56	10s	NM_000201.3
	Reverse	AGCGTAGGGTAAGGTTCTTGC			
FKBP5	Forward	GGCTGGCAGTCTCCCTAAA	56	14s	NM_001145775.3
	Reverse	ATCCCTCTCCTTTCCGTTTGG			
GILZ	Forward	AGCAGCGGGGAGAACAAC	61	24s	NM_198057.3
	Reverse	TCCGGAGGCACTGTGGAA			
TNF	Forward	CCCATGTTGTAGCAAACCCT	56	10s	NM_000594.4
	Reverse	TATCTCTCAGCTCCACGCC			
GRβ	Forward	GAAGGAAACTCCAGCCAGAA	52	14s	NM_001020825.2
	Reverse	GTTGGGATGAAAATCAGATTAATGTG			
GRα	Forward	AAAAGAGCAGTGGAAGGACA	–	–	NM_000176.3
	Reverse	TCGAGCTTCCAGGTTCATTC			
	Probe	ACCTATGTGCTGGAAGGAATGATTGCA			
GRλ	Forward	AAGAGCAGTGGAAGGTAGACA	–	–	NM_001024094.2
	Reverse	GTTCATTCCAGCCTGAAGACAT			
	Probe	ACCTATGTGCTGGAAGGAATGATTGCA			
GRP	Forward	TGCTTCTCTCTTCAGGTTGG	–	–	NM_001204265.2
	Reverse	CTGTTTCTGCCATACCTATTTGTC			
	Probe	CTTATGTCAAAAGCATGAAATATGAAGGCCT			
